# Association of Inpatient Diabetes Specialist Consultation with Clinical Outcomes in Patients with Diabetes Hospitalized for Lower Respiratory Tract Infections: A Retrospective Cohort Study

**DOI:** 10.3390/jcm15187173

**Published:** 2026-09-15

**Authors:** Matthias Domanski, Fabienne Jaun, Joerg Daniel Leuppi, Maria Boesing, Giorgia Lüthi-Corridori

**Affiliations:** 1 University Institute of Internal Medicine, Cantonal Hospital Baselland, 4410 Liestal, Switzerland; 2 Faculty of Medicine, University of Basel, CH-4056 Basel, Switzerland; 3 Harvard T.H. Chan School of Public Health–Executive and Continuing Professional Education ECPE, Boston, MA 02115, USA

**Keywords:** diabetes mellitus, lower respiratory tract infection, inpatient diabetes consultation, diabetes specialist care, length of hospital stay, mortality, multidisciplinary care, retrospective cohort study

## Abstract

**Background**: Diabetes mellitus (DM) is associated with an increased susceptibility to lower respiratory tract infections (LRTIs) and more severe clinical courses, including prolonged hospitalization and increased mortality. During LRTI-related hospitalizations, clinical management often prioritizes the acute respiratory condition, potentially under-recognizing DM-related multimorbidity. We evaluated whether inpatient diabetes specialist consultation is associated with improved outcomes in this high-risk population. **Methods**: We conducted a retrospective cohort study of 677 adults with DM admitted for LRTI. Specialist consultation was defined as involvement of an endocrinology and/or diabetes nurse specialist during index hospitalization. Outcomes included length of hospital stay (LOHS) and 90-day mortality. Multivariable zero-truncated negative binomial regression (LOHS) and logistic regression (mortality) were adjusted for age, sex, Charlson Comorbidity Index (CCI), National Early Warning Score (NEWS), and pre-admission insulin use, hypoglycemic events, and admission laboratory parameters. Inverse probability of treatment weighting (IPTW) based on propensity scores was performed as a sensitivity analysis. **Results**: Among 677 patients, 84 (12.4%) received diabetes specialist consultation. Patients receiving consultation had lower unadjusted 90-day mortality (4.8% vs. 17.5%, *p* = 0.001) but longer median LOHS (9 vs. 7 days, *p* < 0.001). After multivariable adjustment, diabetes specialist consultation was associated with lower 90-day mortality (OR 0.23, 95% CI 0.08–0.68, *p* = 0.008) and longer LOHS (IRR 1.27, 95% CI 1.12–1.44, *p* < 0.001). The results were consistent in IPTW sensitivity analyses. **Conclusions**: In this retrospective cohort of patients with DM hospitalized for LRTIs, inpatient diabetes specialist consultation was associated with lower 90-day mortality, but longer hospital stay. These findings suggest that structured inpatient diabetes specialist involvement may contribute to improved medium-term outcomes in high-risk multimorbid patients. Prospective studies are required to determine causality and evaluate the impact of consultation timing and referral strategies.

## 1. Introduction

Diabetes mellitus (DM) is a highly prevalent chronic condition [1,2] associated with substantial morbidity and mortality worldwide [3,4,5,6]. The global prevalence of DM among adults aged 20–79 years is currently estimated at 11.1%, corresponding to approximately 589 million individuals worldwide [4]. This burden is expected to increase substantially over the coming decades, with prevalence projected to reach 13.0% and the number of affected individuals rising to 853 million by 2050 [3], driven by demographic changes and the growing burden of obesity and metabolic disease. DM is particularly common among patients hospitalized for lower respiratory tract infections (LRTIs), with studies reporting diabetes prevalence ranging from approximately 20% to 30% among patients admitted with community-acquired pneumonia [7,8,9]. Patients with DM face a significantly increased susceptibility and risk of developing LRTIs compared to the general population (OR 1.3–1.4) [10,11,12], with the risk of LRTI-related hospitalization rising substantially in parallel with longer diabetes duration and suboptimal pre-admission glycemic control [13,14,15]. The interaction between DM and LRTIs is complex and multifactorial, involving impaired immune response [16,17], chronic inflammation [18], and metabolic dysregulation [19]. At the cellular level, hyperglycemia weakens host defenses by impairing immune cells (including neutrophils and macrophages), which significantly delays microbial clearance [20,21,22,23]. Concurrently, elevated glucose levels trigger oxidative stress and damage the vascular endothelium, further exacerbating lung inflammation and tissue injury [22,24,25]. Consequently, patients with DM hospitalized for LRTIs often present with higher disease severity, prolonged hospital stays [26], and worse clinical outcomes compared to individuals without diabetes [13,14,27,28,29]. While previous studies have highlighted the role of glycemic control and specific antidiabetic medication schemes (such as SGLT2 inhibitors, GLP-1 receptor agonists, or structured insulin regimens) in influencing outcomes of infectious diseases [30,31,32,33], less attention has been given to the impact of healthcare delivery factors, specifically, structured inpatient interventions that optimize complex diabetes management during acute illness [34,35]. In routine clinical practice, the management of patients admitted with LRTIs frequently prioritizes the acute respiratory condition, potentially leading to the under-recognition and suboptimal management of DM and its associated complications [36]. This knowledge gap is particularly relevant in older multimorbid patients, in whom acute respiratory illness coexists with complex diabetes management challenges, such as high glycemic variability and heightened vulnerability to hypoglycemia.

Specialist inpatient diabetes consultations, including those by endocrinologists and diabetes nurse specialists, represent a modifiable and structured component of care [37]. Such interventions may contribute to improved glycemic management through optimization of antihyperglycemic therapy, management of acute glycemic events (hyperglycemic and hypoglycemic), patient education, and coordination of post-discharge diabetes care. Although specialist diabetes teams are increasingly recommended in inpatient care, evidence regarding their impact on clinically relevant outcomes remains limited and heterogeneous, particularly among patients hospitalized with acute respiratory infections. Thus, studies evaluating whether inpatient diabetes specialist consultation translates into improved outcomes in this high-risk population remain limited [38].

A systematic review of inpatient diabetes specialist nurse services reported reductions in length of stay, although no mortality benefit was observed, while more recent prospective and randomized studies have demonstrated improvements in glycemic control, hypoglycemia, hospital-acquired infections, and other hospital outcomes [39,40,41,42]. Current recommendations similarly acknowledge potential benefits of inpatient glucose-management teams on length of stay and clinical outcomes, without established evidence of a mortality benefit [36]. In contrast, studies examining diabetes in patients with LRTI or pneumonia have mainly evaluated admission glucose and HbA1c as prognostic markers of adverse outcomes and mortality, rather than the potential impact of specialist consultation [13,43,44]. Although a recent randomized respiratory–endocrinology co-management study in patients with COPD exacerbations and diabetes reported shorter hospital stay and fewer readmissions, this population and intervention were not specific to LRTI or pneumonia [9].

Therefore, the aim of this study was to evaluate the association between inpatient diabetes specialist consultation and clinical outcomes in patients with DM hospitalized for LRTIs. Specifically, we assessed its association with length of hospital stay (LOHS) and 90-day mortality.

## 2. Materials and Methods

This manuscript was prepared in accordance with the STROBE (Strengthening the Reporting of Observational Studies in Epidemiology) guidelines.

### 2.1. Study Design and Setting

This retrospective, observational, single-center study was conducted at the Cantonal Hospital of Baselland (KSBL), a Swiss public teaching hospital serving approximately 300,000 people [45].

### 2.2. Study Population

The study population consisted of all consecutive adult patients with DM type 1 and 2 who were hospitalized for LRTI at the Cantonal Hospital Baselland (KSBL) between 1 January 2023 and 31 December 2024. Eligible patients were identified from the hospital’s administrative database based on ICD-10 codes for DM (E10–E14) and LRTI diagnoses (A15, A16, A19, A37, A48.1, A70, J09-J22, J44.0, J44.1, J45, J85.1, U04, U07.1). The administrative database was used for initial case identification, after which potentially eligible records were manually screened according to the predefined inclusion and exclusion criteria. Only the first eligible admission in the time period per patient was included (‘index hospitalization’).

### 2.3. Inclusion and Exclusion Criteria

**Figure 1:**
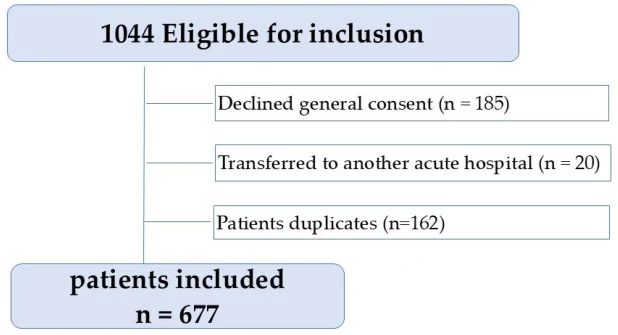
Study flow chart of patient screening and inclusion.

The detailed selection process is illustrated in Figure 1.

Inclusion Criteria

- Age ≥ 18 years;
- ICD-10 codes for diabetes mellitus (E10–E14);
- Discharged from the KSBL between 1 January 2023 and 31 December 2024.

Exclusion Criteria

- Documented decline of general research consent;
- LRTI not contributing to the reason for admission;
- Transfer to or from another acute care hospital with >24 h stay;
- Duplicate patient entries.

### 2.4. Data Collection

Data were extracted from hospital administrative records and electronic medical records, managed using REDCap^®^ (Version 16.1.4)—a web application designed for secure data collection and management. Manual screening was performed to verify eligibility according to the predefined inclusion and exclusion criteria. A randomly selected subset of the data was subjected to a quality control review by a second physician investigator.

### 2.5. Variables and Outcomes

Collected variables included demographics, clinical data at admission, diabetes-specific parameters (e.g., HbA1c), LRTI characteristics (e.g., diagnoses, treatment), and outcomes. The primary exposure, inpatient diabetes specialist consultation, was defined as documented involvement of either an endocrinologist and/or a certified diabetes nurse specialist during the index hospitalization and was analyzed as a binary variable. Admission laboratory parameters were defined as the initial laboratory assessment obtained as part of the routine diagnostic work-up in the Emergency Department. Although no predefined study-specific time window was applied, these measurements are routinely obtained early during the initial assessment, generally within the first hour after presentation and before ward-based treatment. The first available values were used; peak, nadir, or worst values during hospitalization were not considered. Because of the retrospective design, the exact timing of individual emergency interventions relative to blood sampling could not be reliably determined for every patient. HbA1c values were obtained during the index hospitalization or, if available, within the preceding 90 days and were used as a marker of recent chronic glycemic control. Outcomes of interest included LOHS, defined as the total number of days of inpatient care during the index hospitalization, and all-cause 90-day mortality from admission. Post-discharge vital status was ascertained via the hospital electronic database, which receives automated updates from official registries. Follow-up at 90 days was complete with no losses to follow-up.

### 2.6. Statistical Analysis

Statistical analysis was performed using STATA (StataCorp LLC, Version 18.0 BE, College Station, TX, USA).

Descriptive statistics included medians with interquartile ranges (IQRs) for continuous variables and absolute and relative frequencies for categorical variables. Distributions were assessed visually using histograms. Missing data were reported for all variables.

Missing data were addressed using multiple imputation by chained equations (MICE), generating 20 imputed datasets under the assumption of missing at random. The imputation model included all variables used in the regression analyses as well as study outcomes.

Multivariable regression analyses were conducted to assess the association between inpatient diabetes specialist consultation and clinical outcomes. Length of hospital stay was analyzed using zero-truncated negative binomial (ZTNB) regression and 90-day mortality using multivariable logistic regression.

Potential confounders were identified a priori based on clinical relevance and the existing literature. All regression analyses were adjusted for age, sex, Charlson Comorbidity Index (CCI), National Early Warning Score (NEWS), pre-admission insulin use, and hypoglycemic events. Effect estimates are reported as regression coefficients (β), incidence rate ratios (IRRs), or odds ratios (ORs), with corresponding 95% confidence intervals (CIs). A two-sided *p*-value < 0.05 was considered statistically significant.

As a sensitivity analysis, inverse probability of treatment weighting (IPTW) was performed across all M = 20 multiply imputed datasets (*n* = 677) to account for baseline confounding while avoiding selection bias from missing data. Propensity scores for receiving specialist diabetes consultation were estimated using logistic regression including the same baseline covariates used in the primary models to assess the robustness of the primary findings. Propensity score overlap was assessed, and baseline covariate balance before and after weighting was evaluated using standardized mean differences (SMDs).

As an additional sensitivity analysis, the association between specialist diabetes consultation and length of hospital stay (LOHS) was evaluated after excluding patients who died during hospitalization. This analysis was restricted to patients discharged alive. A multivariable zero-truncated negative binomial regression model was fitted using the same baseline covariates as the primary model, with multiple imputation across 20 datasets.

To address potential immortal time bias arising from the timing of inpatient specialist consultation, a Day-4 landmark sensitivity analysis was performed. The landmark cohort was restricted to patients who were alive and remained hospitalized at Day 4. Multivariable logistic regression using multiple imputation was repeated within the landmark cohort to evaluate the robustness of the association between inpatient specialist consultation and 90-day mortality.

## 3. Results

### 3.1. Patient Characteristics

Patient characteristics are displayed in Table 1. Among the 677 patients included in the analysis, a total of 84 patients (12.4%) received inpatient diabetes specialist consultation. Consultation took place a median of 4 days after admission (IQR 2–7), leaving a median of 4 days (IQR 2–7) for diabetes discharge planning. Patients receiving consultation were younger (median 74 vs. 80 years, *p* = 0.001) and had a lower comorbidity burden (CCI ≥ 4: 70.2% vs. 84.5%, *p* = 0.002). The individual CCI components for the overall cohort and stratified by diabetes specialist consultation status are detailed in Supplementary Material (Supplementary Table S1). Overall clinical severity at admission, assessed by the National Early Warning Score (NEWS), was comparable between groups. Standardized differences demonstrate clinically meaningful baseline imbalances for age, comorbidity burden, admission glucose, pre-admission insulin use, and hypoglycemic events. Inflammatory markers were numerically higher in patients receiving diabetes specialist consultation: median CRP was 77 mg/L versus 57 mg/L in patients without consultation (*p* = 0.066), and median leukocyte count was 11.2 G/L versus 9.9 G/L, respectively (*p* = 0.025). Other baseline characteristics, including sex, smoking status, and major comorbidities, were well balanced between groups (Table 1).

### 3.2. Diabetes Characteristics

Most of the overall population had type 2 DM (97.5%), whereas type 1 DM occurred more frequently in the consultation group (11.9% vs. 1%). In the consultation group, 50% of patients had their DM diagnosed more than 10 years prior to the index admission, as opposed to 34.9% in the no-consultation group. Time of diagnosis was not documented in 43.3% overall. The proportion of newly diagnosed cases of DM was 10.7% in the consultation group vs. 3.2% in the no-consultation group (*p* < 0.001), and the proportion of insulin-dependent patients was 50% vs. 30.9% (*p* < 0.001). Around a quarter of patients in the consultation group started on insulin during the index hospitalization. Three-quarters of the total cohort had no long-term diabetes-associated complications (73.4%), while the consultation group tended to experience more of these long-term complications and hypoglycemic events (19.0% vs. 7.4%; *p* < 0.001). Admission glucose in the consultation group was significantly higher, with a median of 12.7 mmol/L vs. 9.2 mmol/L (*p* < 0.001), with only 1.3% of values missing. HbA1c median showed a similar pattern of 8.7% vs. 7% (*p* < 0.001), but 42.8% of the values were missing. For more details please see Table 2.

### 3.3. LRTI Characteristics

Pneumonia was the most common LRTI diagnosis in the overall cohort (51.7%), followed by SARS-CoV-2 infection (30.1%) and COPD exacerbation (12%). The distribution of LRTI diagnoses was comparable between patients who received diabetes specialist consultation and those who did not, with no statistically significant differences observed across all diagnostic categories (see Table 3).

### 3.4. Outcomes

In unadjusted analyses (Table 3), patients receiving diabetes specialist consultation had a longer hospital stay compared to those without consultation (median 9 vs. 7 days, *p* < 0.001). In contrast, 90-day mortality was significantly lower in the consultation group (4.8% vs. 17.5%, *p* = 0.001).

In the multivariable ZTNB regression model adjusted for age, sex, comorbidity burden (CCI), disease severity (NEWS), pre-admission insulin use, hypoglycemic events, and admission laboratory parameters, diabetes specialist consultation was associated with a longer length of hospital stay (IRR 1.27, 95% CI 1.12–1.44, *p* < 0.001, Table 4). Older age, higher comorbidity burden, and hypoglycemic events during hospitalization were also associated with longer hospital stay.

After multivariable adjustment, diabetes specialist consultation was associated with significantly lower odds of 90-day mortality (OR 0.23, 95% CI 0.08–0.68, *p* = 0.008, Table 5). Higher age, greater comorbidity burden, and increased disease severity were independently associated with increased mortality risk.

In the IPTW sensitivity analysis, propensity score distributions showed adequate overlap between patients with and without specialist diabetes consultation. Weighting substantially reduced baseline covariate imbalance. The post-weighting SMDs were ≤0.11 for all covariates, with NEWS and pre-admission insulin use remaining marginally above the conventional 0.10 threshold (SMD = 0.109 and 0.107, respectively), compared with larger imbalances before weighting. Detailed pre- and post-weighting covariate balance is reported in Supplementary Table S2. In the IPTW-weighted outcome models, diabetes specialist consultation remained associated with a longer length of hospital stay (IRR 1.42, 95% CI 1.24–1.63, *p* < 0.001; Appendix A Table A1) and lower 90-day mortality (OR 0.16, 95% CI 0.04–0.67, *p* = 0.012; Appendix A Table A2).

In the sensitivity analysis restricted to patients who survived to hospital discharge (N = 623), specialist diabetes consultation remained associated with a longer hospital stay. In the multivariable zero-truncated negative binomial regression model, specialist consultation was associated with a 29% higher expected length of hospital stay (IRR = 1.29, 95% CI: 1.12–1.49, *p* = 0.001). Among the other covariates, older age and higher NEWS were also independently associated with longer hospital stay, whereas the associations of CCI, pre-admission insulin use, hypoglycemic events, leukocyte count, and glucose at admission were not statistically significant. Full results are reported in Appendix A Table A3.

In the landmark sensitivity analysis addressing potential immortal time bias, 23 early deaths occurred within the first 4 days of admission, representing 3.4% of the total cohort. All 23 early deaths occurred in patients who did not receive specialist diabetes consultation, whereas no early deaths occurred among patients who received specialist consultation (23/593 vs. 0/84; one-sided Fisher’s exact test, *p* = 0.045). After restricting the analysis to the 654 patients who were alive and remained hospitalized at Day 4, specialist diabetes consultation remained significantly associated with lower 90-day mortality (adjusted OR = 0.27, 95% CI: 0.08–0.86, *p* = 0.027). These findings are reported in Appendix A Table A4.

## 4. Discussion

In this retrospective cohort of patients with DM hospitalized for LRTI, inpatient diabetes specialist consultation was associated with significantly lower all-cause 90-day mortality, despite being linked to a longer LOHS after multivariable adjustment. Given the observational design, non-random referral, sparse mortality events in the consultation group, and potential residual time-related confounding, this association should be interpreted as exploratory rather than causal.

### 4.1. Study Population

The analyzed cohort reflects the clinical profile of patients with diabetes mellitus hospitalized for lower respiratory tract infections in routine practice. It is characterized by an older, multimorbid population with a predominance of type 2 diabetes, consistent with real-world inpatient cohorts [46,47].

The high burden of comorbidity is particularly notable, with 82.7% of patients presenting with a CCI ≥ 4, indicating substantial multimorbidity. This is in line with previous studies of hospitalized patients with diabetes [14,31,46,47,48]. However, the median age in our cohort was higher than in studies evaluating diabetes specialist consultation in broader, less selected inpatient populations [49,50,51,52,53].

The distribution of LRTI types, with pneumonia as the leading diagnosis followed by viral etiologies, is consistent with epidemiological data [28,54,55,56] and highlights the ongoing significance of respiratory infections in people with DM [57]. Diabetes-related characteristics, including the predominance of type 2 diabetes and the substantial proportion of missing data on disease duration and chronic complications, are comparable to findings from Swiss registry and primary care data. These patterns likely reflect known documentation gaps, particularly in older patients with long-standing type 2 diabetes [58,59].

Overall, these features suggest that the study population is representative of a high-risk, real-world inpatient cohort, although the advanced age and high comorbidity burden should be considered when extrapolating the findings to younger or less complex populations.

### 4.2. Outcomes

Median LOHS in the overall cohort was comparable to previous European studies of patients with DM hospitalized for respiratory infections [26,60,61,62]. In contrast to prior studies in more heterogeneous and generally younger inpatient populations, where early involvement of diabetes specialist teams has been associated with shorter LOHS [49,50], we observed a significantly longer hospital stay among patients receiving diabetes specialist consultation, even after multivariable adjustment. To address the potential competing effect of early in-hospital mortality (which occurred more frequently in the non-consultation group), we performed a dedicated sensitivity analysis restricted exclusively to patients who survived to hospital discharge (*n* = 623). In the multivariable zero-truncated negative binomial regression model using multiple imputation among discharge survivors, diabetes specialist consultation remained significantly associated with a longer length of stay (adjusted IRR = 1.29, 95% CI: 1.12–1.49, *p* = 0.001; see Appendix A Table A3). Importantly, patients in the non-consultation group had a higher comorbidity burden, reflected by a higher CCI. This suggests that diabetes specialist consultation was not driven by overall comorbidity burden, but rather by clinical prioritization of patients with glycemic instability, complex treatment regimens, or other diabetes-related management difficulties. Although residual confounding by indication cannot be excluded, the persistence of the association after adjustment for comorbidity burden (CCI), disease severity (NEWS), and other relevant covariates suggests that differences in case severity and complexity alone do not explain the observed prolongation of LOHS.

Several explanations may account for the longer hospital stay observed among patients receiving diabetes specialist consultation. Patients in the consultation group also showed numerically higher inflammatory markers at admission, with significantly higher leukocyte counts and higher CRP levels that did not reach statistical significance. This pattern may reflect a greater acute inflammatory or infectious burden, which itself could contribute to longer hospitalization. However, leukocyte count was included in the adjusted analyses, and the association between specialist consultation and longer length of hospital stay persisted after adjustment for this and other baseline covariates. Specialist input may additionally involve treatment adjustment, diabetes education, and discharge planning, which can require additional coordination. Conversely, a longer hospitalization may itself increase the opportunity for a consultation to occur, and residual confounding by diabetes complexity or other unmeasured factors may contribute. The persistence of the association among patients discharged alive reduces, but does not eliminate, concern that early in-hospital death alone explains the finding. Accordingly, the longer LOHS should not be interpreted as a causal effect of specialist consultation.

Consultation occurred a median of 4 days after admission, indicating that exposure timing is an important methodological consideration. Patients had to remain alive and hospitalized long enough to receive specialist input, creating a risk of immortal time bias and reverse causation. The Day-4 landmark sensitivity analysis showed that the association with lower 90-day mortality persisted in the reported landmark cohort (adjusted OR 0.27, 95% CI 0.08–0.86). This analysis reduces concern about bias from very early deaths but does not exclude residual time-related bias, particularly because consultation timing varied and referral was not standardized. Patients were predominantly managed in general internal medicine and geriatric wards under non-endocrinologist care, and the decision to request a diabetes consultation relied on individual clinical judgment. Whether clinicians with differing levels of diabetes expertise provided comparable inpatient glycemic care without formal consultation remains unmeasured.

Overall, 90-day mortality in our cohort was comparable to those reported in previous European studies of patients with diabetes mellitus hospitalized for LRTI [26,46,63,64,65,66].

After multivariable adjustment, diabetes specialist consultation was associated with a significantly lower 90-day mortality. The previous literature on structured inpatient diabetes specialist involvement is limited and heterogeneous and is not directly comparable with the present LRTI population or 90-day endpoint [36,39,67]. The association also remained directionally consistent in propensity score–weighted analyses. IPTW substantially improved measured baseline covariate balance, although NEWS and pre-admission insulin use remained marginally above the conventional absolute SMD threshold of 0.10 (Supplementary Table S2). These analyses reduce concern about measured baseline confounding but cannot address unmeasured confounding, referral bias, or all time-related biases.

Potential mechanisms are hypothesis-generating. Specialist involvement could plausibly influence glycemic management, recognition of hypo- and hyperglycemia, adjustment of antidiabetic therapy, patient education, and discharge coordination; however, the retrospective dataset did not systematically capture the indication, timing, content, or sequence of individual consultation recommendations. Discharge treatment patterns provide limited supportive process information: insulin use was more frequent at discharge in the consultation group, and documented medication changes were more common among patients with available medication-comparison data (Appendix A Table A5). These observations should not be interpreted as demonstrating mediation or causality.

### 4.3. Limitations, Strengths and Future Studies

Several limitations should be considered when interpreting the findings. First, the retrospective single-center design within a hospital network limits causal inference and may reduce generalizability to other healthcare settings.

Second, referral for diabetes specialist consultation was non-random and based on clinical judgment, creating confounding by indication. To mitigate this bias, propensity score–weighted analyses were performed, which confirmed the robustness of the main findings. Nevertheless, residual confounding from unmeasured variables such as pre-admission self-management support, frailty, functional status, cognitive impairment, and social support cannot be excluded. Furthermore, due to the retrospective nature of the study and the lack of standardized electronic order entry or structured reporting for specialist consultations, the specific triggers for requesting a consultation and the precise recommendations provided (e.g., specific nurse education topics vs. specialist physician therapeutic adjustments) could not be systematically captured.

Third, data completeness depended on routine clinical documentation by treating physicians and nurses. This resulted in missing or incomplete variables for established severity scores (e.g., Pneumonia Severity Index). To address missing data, multiple imputation MICE was applied, and the results remained consistent across imputed datasets.

Strengths include a consecutive real-world cohort, complete ascertainment of 90-day vital status, multiple imputation for missing data, multivariable adjustment, and propensity score weighting with reported balance diagnostics. Additional sensitivity analyses examined hospital-stay results among discharge survivors and mortality in a Day-4 landmark cohort. The consistency of direction across these analyses supports the stability of the observed association, while the limitations above preclude causal interpretation. Future studies should validate these findings in prospective, multicenter designs with predefined criteria for diabetes specialist consultation. Systematic collection of frailty, functional status, and cognitive measures would allow a more comprehensive assessment of outcomes beyond mortality and length of stay. Integration of inpatient and outpatient diabetes care pathways may further clarify the long-term impact of specialist involvement in this population. To overcome these retrospective limitations, our research group is currently conducting a prospective randomized pilot study (the INDICATE trial), which will systematically standardize the content, timing, and detailed interventions of inpatient diabetes consultations and prospectively assess their clinical impact.

## 5. Conclusions

In this retrospective cohort study of patients with diabetes mellitus hospitalized for lower respiratory tract infection, inpatient diabetes specialist consultation was associated with a clinically relevant lower rate of 90-day mortality and a longer hospital stay. The mortality association persisted across the reported multivariable, propensity-weighted, and landmark sensitivity analyses; however, the observational design, non-random referral, sparse events in the consultation group, and residual time-related and unmeasured confounding preclude causal inference. The longer hospital stay may reflect diabetes complexity, care processes, reverse causation, or a combination of these factors.

Overall, these findings suggest that inpatient diabetes specialist consultation might be considered an important component of care for high-risk hospitalized patients with diabetes and respiratory infections. These findings should be interpreted in light of the observational design and the possibility of residual confounding. Prospective multicenter studies with predefined referral criteria and systematic assessment of functional status and post-discharge outcomes are required to confirm causality and to further define the role of structured and integrated multidisciplinary diabetes management in this vulnerable population.

## Figures and Tables

**Table 1:** Patient characteristics overall and by presence or absence of consultation.

Patient Characteristic	Overall (*n* = 677)	Missing Values	Consultation (*n* = 84) 12.4%	No Consultation (*n* = 593) 87.6%	*p*-Value
Demographics					
Age [years], median (IQR), range	79 (72–85) 30–103		74 (67–83.3) 30–94	80 (72–86) 45–103	0.001
Female, *n* (%)	289 (42.7%)		39 (46.4%)	250 (42.2%)	0.459
BMI [kg/m^2^], median (IQR)	27.64 (24.3–31.5)	127 (18.8%)	28.53 (24.77–32.1)	27.5 (24.3–31.3)	0.323
Smoking status		390 (57.6%)			0.385
Former smoker, *n* (%)	151 (22.3%)		21 (25.0%)	130 (21.9%)	
Current smoker, *n* (%)	117 (17.3%)		20 (23.8%)	97 (16.5%)	
Comorbidities					
Pulmonary, *n* (%)	280 (41.4%)		33 (39.3%)	247 (41.7%)	
Cardiovascular, *n* (%)	475 (70.2%)		50 (59.5%)	425 (71.7%)	
Arterial hypertension, *n* (%)	574 (84.8%)		72 (85.7%)	502 (84.7%)	0.800
Dyslipidemia, *n* (%)	392 (57.9%)		51 (60.7%)	341 (57.5%)	0.577
Obesity (BMI ≥ 30 kg/m^2^), *n* (%)	189 (27.9%)		30 (35.7%)	159 (26.8%)	
Chronic kidney disease ≥ G3, *n* (%)	299 (44.2%)		36 (42.9%)	263 (44.4%)	0.796
Severity scores					
CCI, median (IQR), range	6 (5–8), 0–15		6 (4–8), 0–13	7 (5–8), 0–15	0.002
NEWS, median (IQR)	5 (3–7)	65 (9.6%)	5 (4–8)	5 (3–7)	0.645
Laboratory parameters at admission					
CRP [mg/L], median (IQR), range	59 (19–143), 0–513	5 (0.7%)	77 (28.5–192.8) 0–513	57 (18–138) 0–490	0.066
Leukocytes [G/L], median (IQR), range	10 (7.9–14)1.6–120	7 (1.0%)	11.2 (8.4–15.4) 2.2–40.4	9.9 (7.8–13.6)1.6–120	0.025

Abbreviations: IQR = Interquartile range; BMI = Body mass index; CCI = Charlson Comorbidity Index; NEWS = National Early Warning Score; CRP = C-reactive protein.

**Table 2:** Diabetes mellitus characteristics, in-hospital glycemia by type of consultation.

Diabetes Mellitus Characteristics	Overall (*n* = 677)	Missing Values	Consultation (*n* = 84) 12.4%	No Consultation (*n* = 593) 87.6%	*p*-Value
Type		1 (0.56%)			<0.001
Type 2 diabetes, *n* (%)	660 (97.5%)		73 (86.9%)	587 (99%)	
Type 1 diabetes, *n* (%)	16 (2.4%)		10 (11.9%)	6 (1%)	
Duration of diabetes diagnosis		293 (43.3%) *			<0.001
DM diagnosis during index case, *n* (%)	27 (4.0%)		9 (10.7%)	19 (3.2%)	
DM diagnosis < 5 years, *n* (%)	60 (8.9%)		6 (7.1%)	55 (9.3%)	
DM diagnosis 5–10 years, *n* (%)	47 (6.9%)		9 (10.7%)	38 (6.4%)	
DM diagnosis > 10 years, *n* (%)	249 (36.8%)		42 (50%)	207 (34.9%)	
Pre-admission DM therapy					<0.001
Dietary and lifestyle change only, *n* (%)	127 (18.8%)		15 (17.9%)	112 (18.9%)	
Oral antidiabetics, *n* (%) ^1^	325 (48.0%)		27 (32.1%)	298 (50.3%)	
Insulin dependency, *n* (%)	225 (33.2%)		42 (50.0%)	183 (30.9%)	
Insulin start during hospitalization, *n* (%)	44 (6.5%)		22 (26.2%)	22 (3.7%)	<0.001
Admission glucose, median (IQR), range	9.5 (7.4–12.5) 0.8–64.4	9 (1.3%)	12.7 (9.4–18.1) 2–64.4	9.2 (7.3–11.9) 0.8–37.1	<0.001
HbA1c [%], median (IQR) ^2^	7.2 (6.5–8.1)	290 (42.8%)	8.7 (7.8–9.9)	7.0 (6.4–7.6)	<0.001
Any hypoglycemic event during index hospitalization < 3.9mmol/L, *n* (%)	60 (8.9%)		16 (19.0%)	44 (7.4%)	<0.001

Abbreviations: HbA1c = Hemoglobin A1c, IQR = interquartile range, DM = diabetes mellitus. ^1^ Non-insulin therapy included Biguanides, SGLT-2 inhibitors, DPP-4 inhibitors, GLP-1 receptor agonists, and Sulfonylureas. ^2^ HbA1c values refer to measurements obtained within 3 months prior to admission or during the index hospitalization, in accordance with ADA recommendations. * Unknown/unrecorded duration of diabetes diagnosis.

**Table 3:** LRTI diagnoses, therapy, and outcomes by type of consultation.

Diagnosis, Therapy, and Outcomes	Overall (*n* = 677)	Consultation (*n* = 84) 12.4%	No Consultation (*n* = 593) 87.6%	*p*-Value
LRTI diagnoses				
Pneumonia, *n* (%)	350 (51.7%)	45 (53.6%)	305 (51.4%)	0.714
SARS-CoV-2, *n* (%)	204 (30.1%)	20 (23.8%)	184 (31.0%)	0.177
COPD exacerbation, *n* (%)	81 (12%)	13 (15.5%)	68 (11.5%)	0.289
Influenza, *n* (%)	73 (10.8%)	5 (6.0%)	68 (11.5%)	0.127
Bronchitis, *n* (%)	35 (5.2%)	5 (6.0%)	30 (5.1%)	0.729
Asthma exacerbation, *n* (%)	16 (2.4%)	4 (4.8%)	12 (2.0%)	0.122
Tuberculosis, *n* (%)	2 (0.3%)	0 (0%)	2 (0.3%)	0.594
Other, *n* (%) ^A^	21 (3.1%)	2 (2.4%)	19 (3.2%)	0.684
LRTI-specific therapy				
Symptomatic treatment only, *n* (%) ^B^	80 (11.8%)	9 (10.7%)	71 (12.0%)	0.738
Oxygen supplementation, *n* (%)	333 (49.2%)	48 (57.1%)	285 (48.1%)	0.119
Antibiotics, *n* (%)	471 (69.6%)	55 (65.5%)	416 (70.2%)	0.383
Systemic corticosteroids, *n* (%)	252 (37.2%)	32 (38.1%)	220 (37.1%)	0.860
Antiviral agents, *n* (%) ^C^	63 (9.3%)	5 (6.0%)	58 (9.8%)	0.258
ICU admission, *n* (%)	92 (13.6%)	14 (16.7%)	78 (13.2%)	0.379
Non-invasive ventilation, *n* (%)	60 (8.9%)	5 (6.0%)	55 (9.3%)	0.316
Invasive ventilation, *n* (%)	11 (1.6%)	2 (2.4%)	9 (1.5%)	0.558
Outcomes				
Length of hospital stay, days	7 (5–12)	9 (6–14)	7 (4–11)	<0.001
90-day mortality, *n* (%)	108 (16.0)	4 (4.8)	104 (17.5)	0.001

Abbreviations: ICU = Intensive care unit, COPD = Chronic obstructive pulmonary disease. ^A^ Included unspecified lower respiratory tract or bronchopulmonary infections, respiratory syncytial virus infections (RSV), bacterial superinfection in RSV infection, viral respiratory infection with bacterial superinfection, exacerbations of recurrent obstructive bronchitis, exacerbation of obstructive pneumopathy, decompensated pulmonary fibrosis, unclear mass with suspected bacterial superinfection, multifactorial dyspnea due to laryngeal carcinoma, and COPD exacerbation. ^B^ Defined as treatment with respiratory physiotherapy, mucolytics, and/or antitussives; ^C^ included Oseltamivir, Zanamivir, and Remdesivir.

**Table 4:** Multivariable zero-truncated negative binomial regression analysis of length of stay including diabetes specialist consultation and other covariates.

Association with Length of Hospital Stay	IRR	95% CI	*p*-Value
Diabetes specialist consultation	1.27	[1.12, 1.44]	<0.001
Age	1.01	[1.01, 1.02]	<0.001
Sex (male)	0.97	[0.87, 1.09]	0.629
CCI	1.02	[1.00, 1.06]	0.038
NEWS	1.01	[0.99, 1.03]	0.286
Insulin dependency pre-admission	1.08	[0.95, 1.23]	0.251
Any hypoglycemic event during index hospitalization < 3.9 mmol/L	1.21	[1.01, 1.47]	0.043
Leukocytes at admission	1.00	[1.00, 1.01]	0.751
Glucose at admission	1.01	[1.00, 1.02]	0.309

Abbreviations: CI = Confidence Interval, CCI = Charlson Comorbidity Index, NEWS = National Early Warning Score, IRR = Incident Rate Ratio.

**Table 5:** Multivariable logistic regression analysis of 90-day mortality including diabetes specialist consultation and other covariates.

Association with 90-Day Mortality from Admission	OR	95% CI	*p*-Value
Diabetes specialist consultation	0.23	[0.08, 0.68]	0.008
Age	1.04	[1.01, 1.07]	0.003
Sex (male)	1.13	[0.70, 1.84]	0.616
CCI	1.34	[1.21, 1.48]	<0.001
NEWS	1.15	[1.06, 1.25]	<0.001
Insulin dependency pre-admission	0.78	[0.46, 1.33]	0.370
Any hypoglycemic event during index hospitalization < 3.9 mmol/L	2.09	[0.91, 4.81]	0.081
Leukocytes at admission	0.98	[0.95, 1.01]	0.167
Glucose at admission	1.03	[0.98, 1.08]	0.230

Abbreviations: CI = Confidence Interval, CCI = Charlson Comorbidity Index, NEWS = National Early Warning Score, OR = Odds Ratio.

## Data Availability

The data supporting the findings of this study are available from the corresponding author upon reasonable request. The data are not publicly available due to ethical and data protection restrictions.

## Supplementary Materials

The following supporting information can be downloaded at: https://www.mdpi.com/article/10.3390/jcm15187173/s1, Table S1: Baseline Charlson Comorbidity Index (CCI) components in the overall cohort and stratified by diabetes counseling status; Table S2: Baseline Covariate Balance Before and After Inverse Probability of Treatment Weighting Across 20 Multiply Imputed Datasets (N = 677).

## Appendix A

**Table A1:** Multivariable zero-truncated negative binomial regression analysis of length of hospital stay after inverse probability of treatment weighting (IPTW) based on propensity scores across imputed datasets (*n* = 677).

Variables	IRR	95% Confidence Interval	*p*-Value
Diabetes specialist consultation	1.42	[1.24, 1.63]	<0.001
Age	1.01	[1.01, 1.02]	<0.001
Sex (male)	1.01	[0.90, 1.14]	0.846
CCI	1.02	[0.99, 1.06]	0.126
NEWS	1.01	[0.99, 1.03]	0.305
Insulin dependency pre-admission	1.10	[0.96, 1.27]	0.180
Any hypoglycemic event	1.25	[1.02, 1.55]	0.036
Leukocytes at admission	0.99	[0.99, 1.01]	0.866
Glucose at admission	0.99	[0.99, 1.01]	0.932

Abbreviations: CCI = Charlson Comorbidity Index, NEWS = National Early Warning Score, IRR = Incidence Rate Ratio.

**Table A2:** Multivariable logistic regression analysis of 90-day mortality after inverse probability of treatment weighting (IPTW) based on propensity scores across imputed datasets (*n* = 677).

Association with 90-Day Mortality from Admission	OR	95% Confidence Interval	*p*-Value
Diabetes specialist consultation	0.16	0.04–0.67	0.012
Age	1.02	0.99–1.06	0.212
Sex (male)	1.13	0.62–2.05	0.699
CCI	1.36	1.23–1.51	<0.001
NEWS	1.18	1.08–1.29	<0.001
Insulin dependency pre-admission	0.68	0.32–1.42	0.300
Any hypoglycemic event	1.59	0.68–3.71	0.288
Leukocytes at admission	0.99	0.95–1.02	0.513
Glucose at admission	1.05	1.00–1.09	0.037

Abbreviations: CCI = Charlson Comorbidity Index, NEWS = National Early Warning Score, OR = Odds Ratio.

**Table A3:** Multivariable zero-truncated negative binomial regression for length of hospital stay among discharge survivors (*n* = 623).

Association with LOHS	IRR	95% Confidence Interval	*p*-Value
Diabetes specialist consultation	1.29	[1.12, 1.49]	0.001
Age	1.01	[1.01, 1.02]	<0.001
Sex (male)	0.98	[0.88, 1.10]	0.766
CCI	1.02	[1.00, 1.05]	0.097
NEWS	1.03	[1.01, 1.05]	0.005
Insulin dependency pre-admission	1.11	[0.98, 1.26]	0.110
Any hypoglycemic event	1.15	[0.95, 1.39]	0.142
Leukocytes at admission	1.00	[1.00, 1.00]	0.849
Glucose at admission	1.00	[0.99, 1.01]	0.714

Abbreviations: CCI = Charlson Comorbidity Index, NEWS = National Early Warning Score, IRR = Incidence Rate Ratio.

**Table A4:** Multivariable logistic regression analysis of 90-day mortality in the landmark cohort at Day 4 (n=654).

Variables	OR	95% Confidence Interval	*p*-Value
Diabetes specialist consultation	0.27	0.08–0.86	0.027
Age	1.05	1.02–1.08	0.003
Sex (male)	1.16	0.69–1.93	0.571
CCI	1.38	1.24–1.53	<0.001
NEWS	1.08	0.99–1.18	0.098
Insulin dependency pre-admission	0.68	0.33–1.21	0.186
Any hypoglycemic event	2.27	1.00–5.15	0.049
Leukocytes at admission	0.99	0.95–1.02	0.451
Glucose at admission	1.05	1.00–1.10	0.064

Note: Estimates were obtained from multivariable logistic regression with multiple imputation (20 datasets) restricted to patients alive and hospitalized at Day 4 post-admission (n=654), excluding early deaths (n=23). Odds ratios for continuous variables are presented per unit increase.

**Table A5:** Comparison of pre-admission vs. discharge diabetes medication schemes and treatment modifications by specialist consultation status.

Diabetes Treatment Transition	Overall Cohort (N = 677)	Consultation (*n* = 84)	No Consultation (*n* = 593)	*p*-Value
Pre-Admission Insulin Use, *n* (%)	225 (33.2%)	42 (50.0%)	183 (30.9%)	<0.001
Discharge Insulin Use, $n\left(\%\right)$	233 (34.4%)	62 (73.8%)	171 (28.8%)	<0.001
Any Diabetes Medication Change at Discharge, $n\left(\%\right)$	114 (33.4%)	28 (47.5%)	86 (30.5%)	0.012
