# Supplementary material for: Association of Inpatient Diabetes Specialist Consultation with Clinical Outcomes in Patients with Diabetes Hospitalized for Lower Respiratory Tract Infections: A Retrospective Cohort Study
Source: J Clin Med. 2026 Sep 15;15(18):7173. doi: 10.3390/jcm15187173 (PMC13607689; doi:10.3390/jcm15187173)
Supplement: Supplementary file 1 [file jcm-15-07173-s001.zip › jcm-4491294-supplementary.pdf]

Supplementary Table S1. Baseline Charlson Comorbidity Index (CCI) components in the overall cohort and stratified by diabetes counseling status.

CCI Comorbidities	overall (n = 677)	counseling (n=84) 12.4%	no counseling (n=593) 87.6%
Myocardial Infarction	243 (35.9%)	24 (28.6%)	219 (36.9%)
Congestive Heart Failure	219 (32.3%)	22 (26.2%)	197 (33.2%)
Peripheral Vascular Disease	109 (16.1%)	14 (16.7%)	95(16.0%)
Cerebrovascular Disease	144 (21.3%)	16 (19.0%)	128 (21.6%)
Dementia	193 (28.5%)	21 (25.0%)	172 (29.0%)
Chronic Pulmonary Disease	181 (26.7%)	24 (28.6%)	157 (26.5%)
Connective Tissue Disease	7 (1.0%)	1 (1.2%)	6 (1.0%)
Peptic Ulcer Disease	18 (2.7%)	3 (3.6%)	15 (2.5%)
Liver Disease			
Mild	19 (2.8%)	2 (2.4%)	17 (2.9%)
Moderate to severe	4 (0.6%)	1 (1.2%)	3 (0.5%)
Diabetes Status			
Without complication	425 (62.8%)	42 (50.0%)	383 (64.6%)
With end organ damage	164 (24.2%)	28 (33.3%)	136 (22.9%)
Hemiplegia	16 (2.4%)	2 (2.4%)	14 (2.4%)
Moderate to Severe Renal Disease	29 (4.3%)	2 (2.4%)	27 (4.6%)
Solid Tumor			
Localised	46 (6.8%)	3 (3.6%)	43 (7.3%)
Metastatic	32 (4.7%)	3 (3.6%)	29 (4.9%)
Leukemia	16 (2.4%)	0 (0.0%)	16 (2.7%)
Lymphoma	17 (2.5%)	1 (1.2%)	16 (2.7%)
AIDS / HIV	0 (0.0%)	0 (0.0%)	0 (0.0%)

Supplementary Table S2. Baseline Covariate Balance Before and After Inverse Probability of Treatment Weighting Across 20 Multiply Imputed Datasets (N=677)

Covariate	Before IPTW: No specialist	Before IPTW: Specialist	SMD before	After IPTW: No specialist	After IPTW: Specialist	SMD after
Age (years)	78.33	73.38	0.422	77.66	78.17	0.048
Sex (male)	57.8%	53.6%	0.086	57.2%	60.0%	0.056
CCI	6.77	5.96	0.302	6.67	6.73	0.025
NEWS	5.24	5.29	0.016	5.19	4.88	0.109
Pre-admission insulin	30.9%	50.0%	0.398	34.6%	39.7%	0.107
Hypoglycemic event	7.4%	19.0%	0.348	8.8%	9.8%	0.033
Leukocytes at admission	11.41	13.02	0.216	11.53	11.06	0.065
Glucose at admission	9.99	15.41	0.745	10.76	10.73	0.005

Note: SMD, standardized mean difference; IPTW, inverse probability of treatment weighting; CCI, Charlson Comorbidity Index; NEWS, National Early Warning Score. Values represent the mean absolute SMD across the 20 multiply imputed datasets. An absolute SMD <0.10 was considered indicative of adequate covariate balance.
